# Bacterial Contamination of the Surgical Site at the Time of Elective Caesarean Section in Belgian Blue Cows—Part 1: Identified by Bacterial Culture

**DOI:** 10.3390/vetsci9120687

**Published:** 2022-12-09

**Authors:** Salem Djebala, Elise Coria, Florian Munaut, Linde Gille, Justine Eppe, Nassim Moula, Bernard Taminiau, Georges Daube, Philippe Bossaert

**Affiliations:** 1 Clinical Department of Ruminants, University of Liège, Quartier Vallée 2, Avenue de Cureghem 7A-7D, 4000 Liège, Belgium; 2 Murphy and Leslie Veterinary Centre (Private Practice), Muckerstaff Granard, N39AN52 Co Longford, Ireland; 3 Department of animal production, University of Liege, Quartier Vallée 2, Avenue de Cureghem 6, 4000 Liège, Belgium; 4 GIGA, Animal Facilities, ULiège, B 34, 4000 Liège, Belgium; 5 Fundamental and Applied Research for Animal and Health (FARAH), University of Liege, 4000 Liege, Belgium; 6 Food Microbiology, Department of Food Sciences, Faculty of Veterinary Medicine, University of Liege, 4000 Liege, Belgium; 7 Faculty of Veterinary Medicine, University of Namur, Rue de Bruxelles 61, 5000 Namur, Belgium

**Keywords:** elective caesarean section, Belgian blue cows, clean contaminated surgery, bacterial contamination, aerobic bacteria, preoperative antibiotic

## Abstract

**Simple Summary:**

To ovoid postoperative complications of elective cesarean section (CS) in Belgian blue cows, practitioners always administrate antibiotics. However, no one knows which bacteria are targeted by this antibiotic therapy. This study aimed to describe the bacteria encountered in the surgical site during elective caesarean section (CS) in order to improve the effectiveness of the used antibiotic therapy and to reduce the occurrence of antibiotic resistance. Bacterial cultures were performed on cotton swabs taken from the visceral and parietal peritoneum of 76 cows during the realization of CS. Bacteria were found in only a quarter of samples, while the other swabs were negative. A total of 32 strains belonging to 18 different species was identified. The majority of isolates were gram-negative aerobic species (62.5%), 34% were gram-positive aerobic bacteria and 3% were anaerobic gram-positive species. Due to the presence of bacteria in the quarter of elective CS, this surgery could be considered as a clean contaminated operation. Antibiotic treatment is advised in clean contaminated surgery. Nevertheless, it must be directed against the most identified bacterial population, in this case aerobic gram-negative strains.

**Abstract:**

To improve the efficacy of preoperative antibiotics used in elective caesarean section (CS), we aimed to identify the bacteria contaminating the surgical site during this surgery. A study was conducted on 76 Belgian Blue cows. Bacteriology was performed on cotton swab sampled from the visceral and parietal peritoneum of each cow during the CS. Most of samples showed a negative culture (55/76; 72.37%), 19/76 (25%) were positive (*p* < 0.0001) and two samples were contaminated. In total, 32 isolates belonging to 18 species were identified. Most of them are aerobic (17/18; 94.44%) and half of them were gram-negative (G-). The most encountered bacteria were *Acinetobacter* sp. (6/32; 18.75%), *Pseudomonas* sp. (4/32; 12.5%), *Aerococcus viridans* (4/32; 12.5%), *Psychrobacter* sp. (3/32; 9.37%), and *Escherichia coli* (2/32; 6.25%). Among the identified isolates, 31/32 (96.87%) were aerobic and 1/32 (3.12%) was anaerobic (*p* = 0.0001). Furthermore, 20/32 (62.50%) strains were G− while 12/32 (37.5%) were gram-positive (G+) (*p* = 0.012). In fact, most of cultured strains were aerobic G− (20/32), 11/32 were aerobic G+ and 1/32 is anaerobic G+ (*p* < 0.0001). In conclusion, most of samples showed a negative bacteriology; however, aerobic G− strains were the most identified in positive swabs. Therefore, preoperative antibiotics should be aimed against these bacteria.

## 1. Introduction

The Belgian blue cattle breed (BBCB) represents 50% of the Belgian cattle populations [1]. Due to foeto–pelvic disproportion, 95% of BBCB calves are born by elective Caesarean section (CS) [2]. Therefore, Belgian rural veterinarians perform an average of 600 CS per year [3,4]. Although CS is a routine surgery in Belgium, post-operative complications are common [5,6,7]. The majority of CS complications result in bacterial infections [6,8,9,10]. In order to prevent post-operative complications, Belgian veterinarians inject great amount of various antibiotic molecules through several administrative means during the CS realisation [3,4]. Unfortunately, the excessive use of antibiotics results in the proliferation of bacterial resistance [11,12]. The antibiotic misuse during elective CS is the direct consequence of the inconsistent guidelines regarding this topic [3,4]. Indeed, the recommendations reported in the scientific literature about the prophylactic antibiotic utilisation during the CS realisation in cows are empirical and not evidence based [6,13,14,15].

As a part of the fight against antibacterial resistance and the overuse of antibiotics in Belgium, the Antimicrobial Consumption and Resistance in Animal (AMCRA) consider bovine CS as a proper contaminated surgery. Therefore, administration of the first line of antibiotic molecules such us penicillin is advised during this operation [16]. However, these guidelines are transposed from human medicine and the general recommendations of surgical antibiotic administration [17,18,19]. Consequently, there is no evidence of their effectiveness or lack thereof in bovine CS.

The surgical prophylactic antibiotic is intended to protect the patients at the moment of CS realisation. Hence, to optimise the prophylactic antibiotic when penicillin suspension is used through intramuscular means, it is advised to inject it one hour before the surgical incision [20]. Moreover, the suitable antibiotic molecule aims to reduce the concentration of germs in the surgical site by targeting the most frequent bacterial populations found during the operation [17,18,19]. Unfortunately, the bacterial population susceptible to be encountered in the surgical site during the bovine CS is not well documented. The only research that has dealt with the topic was achieved by the researchers of Ghent University (Belgium) in 1996. The study had involved 23 cows in which bacterial culture was carried out in foetal fluids sampled during the realisation of CS. The results highlighted the presence of bacteria in 19 out of 23 samples, and the identified bacterial taxa are Staphylococcus, Enterobacteriaceae, Enterococcus, Clostridium and Actinomyces spp. Nevertheless, no antibiotic testing was performed since the bacterial species making up these families were not identified [13]. Without knowing the bacteria that should be targeted by the prophylactic antibiotic, no clear and accurate recommendations can be made.

This study aimed to improve and focus the antibiotic utilisation during the realisation of elective CS in BBCB. In order to achieve this goal, this research should provide the answers for the following questions: (i) Is elective CS really a clean contaminated surgery? (ii) If it is a clean contaminated surgery, what kind of bacteria species could be involved in the surgical site contaminations? (iii) If bacteria species are identified, what is the spectrum of antibiotics that would be active against these bacteria? The long-term goal of this study is to reduce the rate of post-operative complications by improving the antibiotic utilisation according to targeted species. Furthermore, due to the important number of CS achieved every year in Belgium [1], the enhancement of prophylactic antibiotic treatment will reduce the antibacterial consumption as well as the development of bacterial resistance [12,16].

## 2. Materials and Methods

All procedures received the approval of the Ethical Committee of Liège University (File number 2142). The cows’ owners were informed about the study and gave their consent.

### 2.1. Animals Description and Samples Utilisation

The data of this study were collected between February and June 2020. Two swabs were taken during the elective CS realisation from the peritoneum of seventy-six healthy BBCB that did not receive any treatment for at least seven months beforehand. Cows came from 25 distinct farms in the province of Luxembourg (Wallonia, Belgium). The first swab was kept at 4 °C and dispatched within the day to the regional laboratory of animal health and identification (ARSIA) (Ciney, Belgium) to achieve bacteriology. Results of bacterial cultures are displayed in detail in this research, part 1 of the study (Bacterial contamination of the surgical site at the time of elective caesarean section in Belgian blue cows—Part 1: Identified by bacterial culture). The second sample was immediately frozen and kept at −80 °C to achieve microbiota determination (16 rDNA amplicon sequencing) in the laboratory of Food Microbiology of Liege University. The amplicon sequencing results are displayed in the part 2 of this study (Bacterial contamination of the surgical site at the time of elective caesarean section in Belgian blue cows—Part 2: Identified by 16Sr DNA sequencing).

### 2.2. Caesarean Section Realisation

The moment of CS realisation was decided upon the vaginal palpation performed by the farmer and confirmed by the vet. Elective CS was carried out in each cow between the moment of the passive phase of cervical dilatation, in which the cervix is sufficiently opened to admit two to four fingers and the phase of full cervical dilatation with intact foetal membranes [21,22]. The CS were performed following the recommendations of Kolkman et al. (2007) [14] and Kolkman et al. (2010) [15]; however, small practical modifications were implemented, since CS were carried out in the field. All CS were performed by a 7-years-experienced practitioner. The surgery was always carried out in an upright cow imbedded in its usual place among the other cows in the late stage of pregnancy.

#### 2.2.1. Cow Preparation for Caesarean Section

The left flank is abundantly washed from the 10th rib till after the tuber coxae, using dish soap. Then, an ample area of the flank is shaved and completely cleaned by clear water. A vertical straight local anaesthesia of 40 to 50 cm is performed intramuscularly in the left flank, 10 cm ventrally of the lumbar vertebrae and 10 cm caudally to the last rib. The anaesthesia is performed using 80 mL to 120 mL (3200 mg to 4800 mg) of procaine with adrenaline (Procain hydrochloride^®^, Inovet, Arendonk, Belgium).

#### 2.2.2. Operator and Material Preparation

Before the onset of the operation, the surgical material (scalpel with blade, different pairs of scissors, a number of blood vessels clamps and a number of cutting and round needles) is soaked in diluted chlorhexidine (0.5%) (Ecutan 5%^®^, Ecuphar, Antwerp, Belgium). The surgeon wears a disposable plastic apron and long plastic gloves (covering the hands and arms) doubled by latex gloves (covering the hands). The surgery can start after disinfecting the operator’s hands and the surgical site using towels soaked in the diluted chlorhexidine (0.02%) (Ecutan 5%^®^, Ecuphar, Antwerp, Belgium).

#### 2.2.3. Surgical Procedure

The incision of skin, muscles and parietal peritoneum is performed over 30 to 45 cm along the line of local anaesthesia. When the abdominal cavity is reached, the surgeon seizes the pregnant uterine horn and guides it to the abdominal wound. The uterus is incised along the large curve of the pregnant horn, wide enough to exteriorize the calf. Once the calf is exteriorised, the vet verifies the absence of bleeding and then sutures the uterus in two layers using monofilament synthetic absorbable thread (Surgicryl^®^, SMI AG, St.Vith, Belgium). The first layer is closed by a simple continuous pattern suture while the second is performed by a modified Cushing suture. After that the uterus is replaced inside the peritoneal cavity. The muscular sutures of the abdominal wall were performed in two layers (peritoneum with transverse muscle and internal oblique with external oblique muscle) using polyfilament synthetic absorbable thread (Glycofil^®^, Génia, Saint-Hilain de Chaléons, France). The skin was closed by a simple continuing pattern suture using non-absorbable synthetic polyfilament thread (Supramid^®^, SMI AG, St.Vith, Belgium). In order to avoid any interference between the antibiotic and the results of bacterial culture, 22,000 IU/kg of penicillin (Peni-Kel^®^, 300,000 IU/mL, Kela Laboratoria, Hoogstraten, Belgium) were administered through intra-muscular means in the neck at the end of the surgery. The cows involved in this study were closely monitored by the owner to identify post-operative complications for three months following the CS. Nevertheless, none of these cows have showed any post-operative complications.

### 2.3. Samples Taken

Samples were carried out just after replacing the sutured uterus in the abdominal cavity. At this moment a sterile swab (STERILE R^®^, Piove di Sacco, Italy) is taken by swiping a long line of 10 cm, 2 cm in parallel to the uterus suture (visceral peritoneum of the uterus) and a 10 cm long line perpendicular and below the abdominal incision (parietal peritoneum). The sample intended for the bacteriology was kept at 4 °C and dispatched within the day to the laboratory.

### 2.4. Bacterial Culture and Laboratory Analysis

The sampled swabs were used for aerobic and anaerobic bacteriological culture. Samples for aerobic culture were grown on Columbia agar, Gassner and Columbia/Nalidixic acid agar media (Thermo Fisher Scientific, Brussels, Belgium) at 37 ± 2 °C. Samples for anaerobic culture were grown under anaerobic conditions on Schaedler medium (Thermo Fisher Scientific, Brussels, Belgium) at 37 ± 2 °C. Two readings of each medium were performed at 18 to 24 h and 36 to 48 h of incubation. Bacterial identification of positive culture was performed by the Maldi Biotyper^®^ (Bruker Daltonics, Bremen, Germany). A direct transfer method with on-target formic acid treatment was performed. Results were assessed using the manufacturer’s standard criteria. In fact, species were assigned for scores of ≥2.0, and genera were assigned for scores of ≥1.7 but <2.0. If scores were lower than 1.7, no identification was assigned. The cutoff scores were altered by reducing the standard 2.0 species cutoff to 1.9, 1.8 and 1.7 and the standard 1.7 genus cutoff to 1.6 and 1.5 [23]. The culture was considered “negative” if no bacterial growth was observed, “positive” when one to four bacteria were found and “positive contaminated” when more than four types of bacteria were cultured.

### 2.5. Statistical Analysis

Statistical analyses were performed using SAS (2001). Descriptive analysis was carried out for the number of samples taken in each farm and the number of bacteria found in each positive sample.

Data distribution was checked with a Shapiro–Wilk test, and the median was used to display non-normal distributed results.

Chi-square and Fisher tests were used to compare between the number of positive and negative samples in the bacteriology, the number of bacteria found in positive samples, the number of aerobic and anaerobic species and strains, the number of gram-positive and gram-negative species and strains, the number of gram-positive aerobic, gram-positive anaerobic, gram-negative aerobic and gram-positive anaerobic strains, the number of farms with none positive sample and those showing at least one positive culture. The procedure “Proc Freq” in SAS was used for all statistical analyses; the cutoff of significance was fixed at *p* < 0.05.

## 3. Results

In total, 76 Belgian blue cows were sampled during the CS’s realisation. These cows come from 25 different farms located in the south of Belgium.

Bacteriology was negative in the majority of sampled cows (55/76; 72.37%), it was positive in only 19/76 (25%) samples (*p* < 0.0001), while two samples were considered as contaminated since more than four bacteria species were grown in each one. The number of bacteria identified in the positive samples varied between one and four with a median of one bacterium by sample. Among the 19 positive samples, 1 bacterium was identified in 10/19 (52.63%), 2 bacteria were cultured in 6/19 (31.57%), 3 were found in 2/19 (10.53%) and 4 in 1/19 (5.26%) samples (*p* = 0.006).

**Figure 1:**
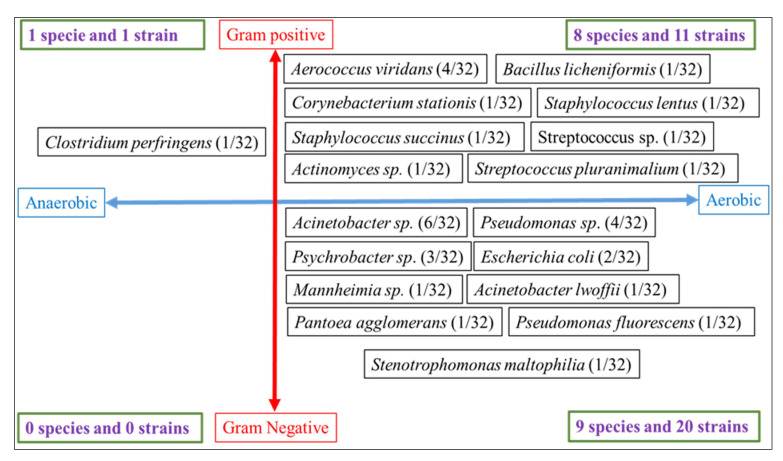
Features of the bacterial isolates (growing in aerobic vs. strict anaerobic environment and gram-positive vs. gram-negative strains) identified in the positive samples (19/76) [24].

In total, 32 bacterial isolates belonging to 18 species were identified. The majority of these species were aerobic (17/18; 94.44%) and only 1/18 (6.56%) was strictly anaerobic (*p* = 0.0002). In contrast, half (9/18; 50%) of identified species were gram-negative, while the other half (9/18; 50%) belonged to gram-positive species (*p* = 1). Among the 32 identified isolates, 31/32 (96.87%) were aerobic and only 1/32 (3.12%) was strict anaerobic (*p* = 0.0001). Furthermore, 20/32 (62.5%) were gram-negative while 12/32 (37.5%) strains were gram-positive (*p* = 0.012). In fact, most of the cultured strains were aerobic gram-negative (20/32), some of them were aerobic gram-positive (11/32) and 1/32 is strict anaerobic gram-positive (*p* < 0.0001). The most encountered bacteria species were *Acinetobacter* sp. (6/32; 18.75%), *Pseudomonas* sp. (4/32; 12.5%), *Aerococcus viridans* (4/32; 12.5%), *Psychrobacter* sp. (3/32; 9.37%) and *Escherichia coli* (2/32; 6.25%); the other bacteria species were only identified once. The details of the culture results are displayed in the Figure 1 and Table 1.

**Figure 2:**
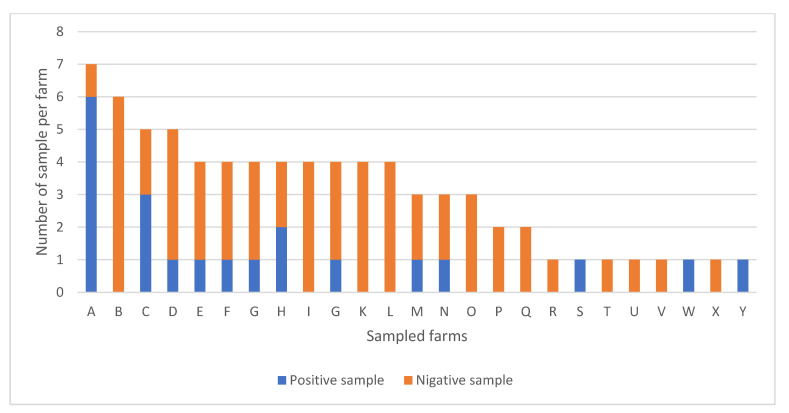
Results of bacterial cultures (positive vs. negative) according to the originate farm of samples.

According to the farm, one to seven samples were taken from each one, with a median of three samples by farm. Although no equal number of samples was taken in the different farms, the investigation of the bacterial culture’s results shows that some farms have non-positive cultures, while most of samples were positive in the others. In fact, the most sampled farm showed 6/7 positive cultures while in the second most sampled one, no positive culture (0/6) was observed. Among the 25 sampled farms, 13 (52%) showed at least one positive culture compared to 12/25 (48%) in which all the cultures were negative (*p* = 0.77). The results of bacterial cultures according to sampled farms are displayed in detail in the Figure 2.

## 4. Discussion

Despite the high amount of elective CS performed each year in Belgium [3,4], and the great risk of post-operative complications [5,6,7,10,25], there are no evidence-based guidelines concerning the prophylactic antibiotic treatment during the realisation of elective CS. This study presents the largest and most recent dataset concerning bacterial contamination during the CS realisation. The sole other study which previously treated this topic was performed on 23 cows [13]. Although this study paved a way for our research, several inaccuracies were noticed. In fact, it is not clear whether CS was elective or performed after obstetrical manipulations and foetal membrane interruption. Moreover, the authors did not highlight bacterial species involved in the contamination; they were focused only on families of bacteria. Finally, the number of cows involved in the study is too low to draw a solid conclusion. All these imprecisions were avoided in the current research, since the samples were taken from a significant number of cows undergoing elective CS. Furthermore, the laboratory analysis were conducted to highlight the bacteria species involved in CS site’s contamination.

The sampling was performed near the uterus suture, allowing the evaluation of the potential exogenous contamination [13,26] since this site is exteriorised during the uterus closure and it is the most manipulated spot by the practitioners within the operation [14,15]. The same swab swept the parietal peritoneum located below the abdominal wound, since after the uterus incision the foetal fluids accumulate in this area [14,15], in order to evaluate the endogenous contamination of the CS [13,26].

Although most of the samples showed a negative culture, 25% displayed a positive result; in addition to that, several bacteria species were cultured in numerous positive samples. The number of positive cultures may even be an underestimation of the true presence of bacteria due to the limited sensibility of bacteriological culture [27]. The results of this study provide the evidence that elective CS is clean contaminated surgery. Consequently, it confirms the need of prophylactic antibiotic usage during the realisation of this operation [16].

In contrast to the rapport of Mijten and collaborators (1996) [13], in which the most identified bacteria belonged to anaerobic and gram-positive families, raising the assumption of endogenous contamination of CS, the results of our study highlight mainly gram-negative aerobic bacteria, which fit with an exogenous contamination of the surgery. In fact, almost the totality of identified bacteria in the current study are ubiquitous and from environmental origin [24]. The great risk of environmental contamination is due to the realisation of CS in highly infectious pressure conditions [7]. These conflicting results could be explained by various hypotheses. In fact, the lack of anaerobic bacteria in our study could be linked to the integrity of foetal membranes before the CS realisation, preventing the spread of the vaginal flora toward the surgical sit. Nevertheless, the foetal membranes were probably ruptured in the study of Mijten and collaborators (1996) [13], resulting in the contamination of the foetal fluids and the surgical site by the anaerobic vaginal flora. Moreover, the different sampling methods promote the identifications of anaerobic bacteria (endogenous contamination) when the sampling was performed by the puncture of foetal fluid before the uterus incision [13]. In contrast, samples taken in exteriorised and heavily manipulated visceral peritoneum of the uterus support the identification of the environmental bacteria. In addition to that, the research of Mijten and collaborators (1996) [13] was performed in clean conditions at the clinic of Ghent University, decreasing the risk of exogenous contamination. In contrast, CS in our study were performed in field conditions, with a considerable risk of environmental contamination. Finally, the low number of identified anaerobic bacteria in the current study could be related to the difficulties faced in culturing this kind of bacteria species. In fact, anaerobic culture is very demanding; it needs a special medium, anaerobic environment and quick dispatching of samples to the laboratory [28].

On the one hand, if the goal of preoperative prophylactic antibiotics is to target the most identified bacterial population during the surgery [17,18,19], our results show that gram-negative aerobic bacteria would be the main focus of the prophylactic antibiotic treatment during the elective CS. In contrast, the most administered antibiotic during elective CS in the Belgian field is penicillin [3,4]. However, the spectrum of this molecule is directed toward the gram-positive and anaerobic species [29], indicating that the administration of penicillin may reach around one-third of the identified strains. The administration of antibiotics targeting gram-negative bacteria such as gentamycin, streptomycin and colistin [29] could be more efficient, since it may reach around two-thirds of the identified strains. Nevertheless, these molecules are not registered for adult cows in Belgium [29] and their utilisation is protected by the AMCRA, since only first line antibiotic are allowed for this kind of surgery [16]. On the other hand, if prophylactic antibiotics are meant to target the bacteria involved in post-operative complications such as *Escherichia coli* or *Pseudomonas* sp. [9,30,31] found in the current study, antibiotics with gram-negative spectrum might be more adapted [31], since these bacteria are not susceptible to penicillin [29]. Although the majority of identified bacteria species in this research are not reported in post-operative complications, it would be useful to assess their pathogenicity in order to get a better insight into the management of prophylactic antibiotic treatments during the elective CS.

Through this study, we figured out that environmental bacteria are the biggest source of elective CS contamination. Therefore, to reduce the rate of post-operative complications and antibiotic consumption [16], the main focus should be put on the scrub and disinfection methods of the operator and the surgical site. Moreover, to decrease the risk of contamination, elective CS should be performed in a clean area such as an adapted calving room [14], rather than elsewhere in the barn as performed in our study and reported in the survey of Hanzen and collaborators (2011) [7]. The fact that some farms show 100% of negative culture and others display a high number of positive samples in this research might be related to the cleanliness of the stable and the atmosphere in which CS were performed.

Although the current research has brought answers for several questions and raises key points to reduce the contamination and improve the antibiotic usage during the CS, our study would be more complete if we had performed antibiotic susceptibility for each identified strain. This would unveil with precision the most adequate antibiotic molecule against the targeted bacterial population. Moreover, due to the limited sensibility of bacteriological culture [27], the combination of bacteriology with other methods based the acid deoxyribonuclease (DNA) sequencing, such as 16S rDNA amplicon sequencing, would be required to reveal all the bacteria species potentially present during the elective CS and not found by bacterial culture [32,33]. Nevertheless, this approach is the subject of the second part of this research (Bacterial contamination of the surgical site at the time of elective caesarean section in Belgian blue cows–Part 2: Identified by 16Sr DNA sequencing).

## 5. Conclusions

In conclusion, our study provides evidence that elective CS is a clean contaminated surgery and the majority of the identified bacteria come from the environment (exogenous contamination); the most involved strains are aerobic gram-negative bacteria. These bacteria should be targeted by preoperative antibiotics during the realisation of elective CS. Moreover, these results should be taken into account when forming new guidelines about prophylactic antibiotic administration during elective CS.

## Figures and Tables

**Table 1:** Bacteria species and bacteria isolates identified in each positive sample (19/76 positive samples and 2/76 contaminated samples), no bacterium was identified in non-displayed samples (55/76) in the table.

Origin of the Sample (Farm)	Positive Samples	Number of Species in Each Sample	Bacterium 1	Bacterium 2	Bacterium 3	Bacterium 4
A (6/7 positive)	1	1	*Pseudomonas* sp.	*/*	*/*	*/*
	2	1	*Psychrobacter* sp.	*/*	*/*	*/*
	3	1	*Aerococcus viridans*	*/*	*/*	*/*
	4	2	*Aerococcus viridans*	*Psychrobacter* sp.	*/*	
	5	2	*Pseudomonas* sp.	*Stenotrophomonas maltophilia*	*/*	*/*
	6	2	*Acinetobacter* sp.	*Psychrobacter* sp.	*/*	*/*
C (3/5 positive)	14	2	*Escherichia coli*	*Acinetobacter* sp.	*/*	*/*
	15	2	*Acinetobacter* sp.	*Pseudomonas fluorescens*	*/*	*/*
	16	More than 4	*/*	*/*		
D (1/5 positive)	19	2	*Actinomyces* sp.	*Streptococcus* sp.	*/*	*/*
E (1/4 positive)	24	1	*Acinetobacter* sp.	*/*	*/*	*/*
F (1/4 positive)	28	1	*Bacillus licheniformis*	*/*	*/*	*/*
G (1/4 positive)	32	1	*Mannheimia* sp.	*/*	*/*	*/*
H (2/4 positive)	36	3	*Acinetobacter lwoffii*	*Aerococcus viridans*	*Pseudomonas* sp.	*/*
	37	3	*Aerococcus viridans*	*Pantoea agglomerans*	*Staphylococcus lentus*	*/*
G (1/4 positive)	44	More than 4	*/*	*/*	*/*	*/*
M (1/3 positive)	56	1	*Clostridium perfringens*	*/*	*/*	*/*
N (1/3 positive)	59	1	*Streptococcus pluranimalium*	*/*	*/*	*/*
S (1/1 positive)	70	1	*Pseudomonas* sp.	*/*	*/*	*/*
W (1/1 positive)	74	4	*Acinetobacter* sp.	*Corynebacterium stationis*	*Escherichia coli*	*Staphylococcus succinus*
Y (1/1 positive)	76	1	*Acinetobacter* sp.	*/*	*/*	*/*

## Data Availability

All data are available in the manuscript.
